# proMAD: semiquantitative densitometric measurement of protein microarrays

**DOI:** 10.1186/s12859-020-3402-4

**Published:** 2020-02-24

**Authors:** Anna Jaeschke, Hagen Eckert, Laura J. Bray

**Affiliations:** 1 Institute of Health and Biomedical Innovation, Queensland University of Technology, Kelvin Grove, Queensland, 4059 Australia; 2 School of Mechanical, Medical and Process Engineering, Science and Engineering Faculty, Queensland University of Technology, Kelvin Grove, Queensland, 4059 Australia; 3 Institute for Materials Science and Max Bergmann Center of Biomaterials, Technische Universität Dresden, Dresden, Germany; 4 Dresden Center for Computational Materials Science (DCMS), Technische Universität Dresden, Dresden, Germany

**Keywords:** Membrane antibody array, Protein microarray, Densitometry, Web application, Python

## Abstract

**Background:**

Protein microarrays are a versatile and widely used tool for analyzing complex protein mixtures. Membrane arrays utilize antibodies which are captured on a membrane to specifically immobilize several proteins of interest at once. Using detection antibodies, the bound protein-antibody-complex is converted into visual signals, which can be quantified using densitometry. The reliability of such densitometric assessments depends on a variety of factors, not only sample preparation and the choice of acquisition device but also the selected analysis software and the algorithms used for readout and processing data. Currently available software packages use a single image of a membrane at an optimal exposure time selected for that specific experimental framework. This selection is based on a user’s best guess and is subject to inter-user variability or the acquisition device algorithm. With modern image acquisition systems proving the capacity to collect signal development over time, this information can be used to improve densitometric measurements. Here we introduce *proMAD*, a toolkit for protein microarray analysis providing a novel systemic approach for the quantification of membrane arrays based on the kinetics of the analytical reaction.

**Results:**

Briefly, our toolkit ensures an exact membrane alignment, utilizing basic computer vision techniques. It also provides a stable method to estimate the background light level. Finally, we model the light production over time, utilizing the knowledge about the reaction kinetics of the underlying horseradish peroxidase-based signal detection method.

**Conclusion:**

*proMAD* incorporates the reaction kinetics of the enzyme to model the signal development over time for each membrane creating an individual, self-referencing concept. Variations of membranes within a given experimental set up can be accounted for, allowing for a better comparison of such. While the open-source library can be implemented in existing workflows and used for highly user-tailored analytic setups, the web application, on the other hand, provides easy platform-independent access to the core algorithm to a wide range of researchers. *proMAD’s* inherent flexibility has the potential to cover a wide range of use-cases and enables the automation of data analytic tasks.

## Background

Protein microarrays provide a versatile platform for the large-throughput analysis of numerous proteins present in a complex mixture based on the highly specific interactions between antibodies and antigens [1, 2]. The "sandwich assay," a multiplexed format of the Enzyme-linked Immunosorbent Assay (ELISA), is a widely used type of microarray. A variety of specific capture antibodies are immobilized on an array membrane. The sample, a blend of various proteins, is incubated with the membrane, and each target protein is trapped by a matching antibody capturing dozens of targets in parallel. A second antibody, detection or reporter antibody, is then employed to generate a chemiluminescent or fluorescent signal which can be detected using a film or, nowadays more commonly used, a CCD camera. While fluorescent dyes allow for multicolor detection systems, enzyme-based methods provide a significant improvement of sensitivity due to the amplification of the signal [2]. The most frequently used enzyme is horseradish peroxidase, which catalyzes the reaction of luminol with H_2_O_2_, thereby generating light [3, 4].

The signals on the array membrane are visible as bright spots generated by the protein-antibody-detection-reagent-complex on a dark background. The signal intensity of a spot and the abundance of the target protein are linked. Besides the quantitative assessment of the spots, presence vs. absence, the optical density of the signals can be quantified by densitometry. This quantification technique is also used in other immunoblot-based assays such as Western Blots [5]. Numerous aspects contribute to the reliability and reproducibility of densitometric assessments. Besides sample preparation techniques and the choice of acquisition device, the algorithms used for data processing are essential factors to consider [5–7]. Several programs are available for the quantification of immunoblots, some are linked to an acquisition software package, and others are specific for certain assay types. The method of densitometric measurement and the approaches for background subtraction vary between applications and details of the algorithms are often not openly accessible. Commonly, the densitometric readout is performed on an image at a particular chosen exposure time. This exposure time is determined either by the researcher or an algorithm in the acquisition software. Usually, some form of background subtraction method is applied. The details of these algorithms are determined by the applied software package. As there are no standardized protocols available, immunoblot densitometry has been described as based on traditions and guesswork [5].

Modern image acquisition systems are improving with regards to sensitivity, user-friendliness, and algorithms finding the optimal exposure time. These machines also provide the ability to capture images over a range of exposure times, allowing for the recording of signal development over time and capturing dynamic changes. Utilizing this information has the potential to improve densitometric quantification approaches for immunoblots. However, currently available analysis tools are not equipped for the large-throughput tasks required for time-based analytics. At present, such image analysis would require time-intensive manual handling. To our knowledge, there is no analysis tool available which specifically employs the dynamic development of optical signals for protein microarray membrane analysis.

Here we introduce *proMAD*, a toolkit for analyzing protein microarrays and a novel systemic analytical concept for the quantification of optical signals detected on the membrane. Utilizing the information of dynamic signal change over time combined with the reaction kinetics, *proMAD* allows us to model the signal development for each membrane. Thereby, the signal quantification does not rely on the information obtained from a single image acquired at a particular exposure time, which might not be optimal for every membrane in a set of membranes. This individual, self-referencing approach allows for a better comparison of different membranes from the same kit within a given experimental framework.

With the *proMAD* open-source library, the core algorithm can be implemented in highly customizable workflows which includes user-defined membrane layouts. A simple, and platform-independent access to the toolkit is provided by the *proMAD* web application. Thereby, the toolkit is accessible for a wide range of users and use-cases such as various membrane types.

## Implementation

The core algorithm used in *proMAD* consists of three main parts. Firstly, the steps to align the raw image sets are presented. Secondly, an approach to estimate the background light level in a stable manner is described. Finally, the correlation between reaction progression and light production over time is demonstrated. The detailed experimental procedures for obtaining the processed images are described in the Supplementary information.

### Image alignment

Exact alignment of the image sets is imperative to ensure correct signal readout and to generate reproducible results. Multiple causes can affect image alignments such as rotation, warp, or stretching. For example, due to the dampness of the membrane, it can lay skewed on the recording tray. In other cases, the position might not remain constant over time. To correct the membrane position, basic computer vision is employed.

**Fig. 1:**
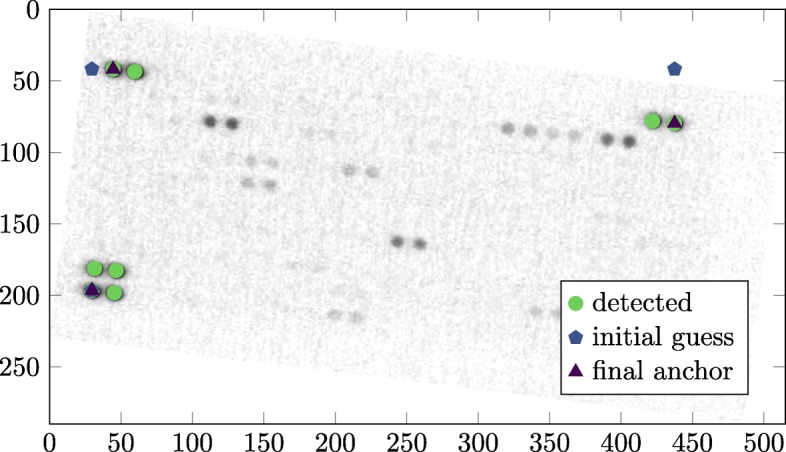
Image alignment. Example membrane image which is not aligned correctly. The detected bright spots are displayed as circles (). The initial guesses *G*_1_,*G*_2_,*G*_3_ of the anchor points are represented as diamonds (), and the final anchors as triangles (). The image of the membrane is inverted for clarity

To align the images, the software relies on the ordered structure of the analytic spots on the membrane. The bright “reference spots” on the edge of the membranes are convenient markers for adjustment. Initially, the images provided by the user either need to be already roughly rotated upright or a rotation parameter needs to be passed to the library. Subsequently, the software finds the anchor points automatically by extracting the contours of the brightest spots. For this purpose, the two-dimensional version of the marching cube algorithm is used [8]. Thereby, the contours are searched at 60% of the maximum gray value found in the raw image and for each contour, a central point *C* is calculated (Fig. 1 ). From the collection of central points, three points are selected to generate the first guess *G* (Fig. 1 ) of the anchor point positions as outlined in Eqs. 1 to 3.

1 $$\begin{array}{*{20}l} G_{1} &= \left[\min(C_{\mathrm{X}}), \min(C_{\mathrm{Y}})\right] \end{array} $$

2 $$\begin{array}{*{20}l} G_{2} &= \left[\min(C_{\mathrm{X}}), \max(C_{\mathrm{Y}})\right] \end{array} $$

3 $$\begin{array}{*{20}l} G_{3} &= \left[\max(C_{\mathrm{X}}), \min(C_{\mathrm{Y}})\right] \end{array} $$

For each estimated point, the contour center *C* with the shortest distance to the initial guess *G* is selected as an anchor point (Fig. 1 ).

It is important to note that this part of the algorithm would need modification to support membranes that have an alternative layout of “reference spots”. Three references points are required to implement a full alignment.

Three tests are applied to ensure that the selected points are meaningful prior to the modification of the image: First, the distance ratio between the three points is compared to the values expected for the particular membrane type. Second, the angle formed by the points and third, the distance between the initial guess and the reference points are checked. The expected values might differ for each membrane type and are determined in the array configuration file. If the checks are within the given tolerances, the image is warped and trimmed. The overall dimension is chosen in such a way, that each spot is centered in a 30-pixel square. This particular value can be changed without altering the results of the presented methods in this paper.

### Background estimation

During the recording of the image sets, the sensor collects signals that are not related to the analytical reaction in the corresponding spot. Light originating from an imperfect enclosure or reflections of the whole membrane is recorded over time. Additionally, noise generated by the sensor accumulates as well. Quantifying this background noise is essential to improve the quality of the data extraction and can also be used as a reference point to compare single images with unknown exposure times, that were measured in the same setup.

**Fig. 2:**
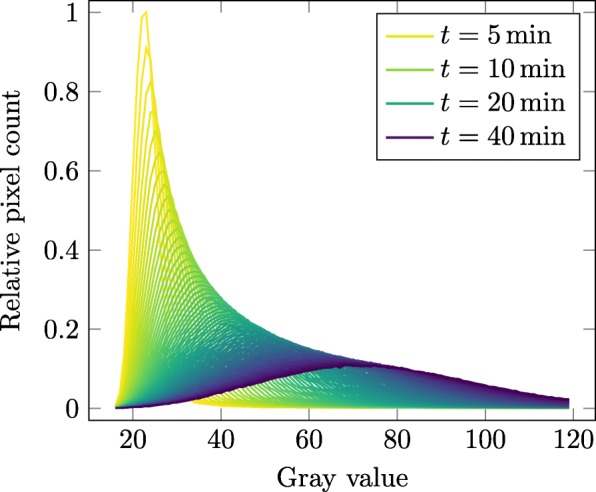
Background noise distribution. Evolution of the background noise distribution over time for a single membrane. The grayscale precision is reduced to 2048 steps for histogram representation

**Fig. 3:**
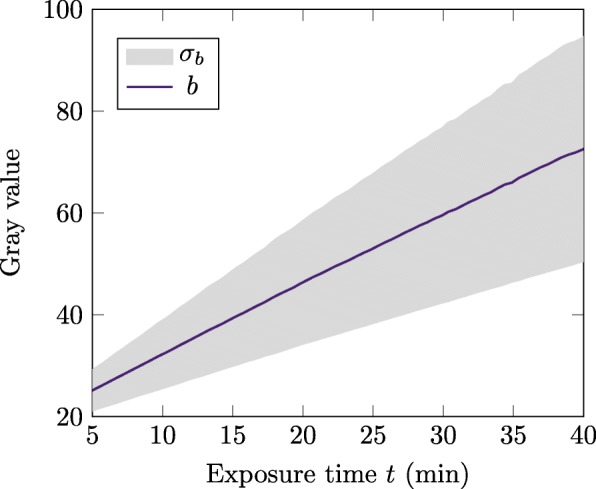
Background. Background gray value development *b* over time *t*. The gray area indicates the standard deviation ±*σ*_*b*_

In this work, an approach based on the normal distribution of the background noise was used. The vast majority of the membranes can be considered dark. Therefore, an extensive sample range is available. To adjust for different input formats, all imported images are internally scaled to be in the range between zero and one. However, the process of generating the background value *b* is demonstrated in Figs. 2 and 3 on an unscaled 16 bit example, to visualize the steps more concisely.

Initially, the image data of each time step is treated as a simple collection of values. Next, the dataset is reduced from of 65 536 to 2048 levels of precision to enable better statistical analytics. The highest peak (see Fig. 2) in these 2048 bins represents the first guess of the background gray value *b*_G_. All values higher than 2*b*_G_ are discarded and as such are not considered part of the background noise. This cutoff ensures that the light originating from the chemical reaction does not alter the background level quantification. The remaining values are fitted to a normal distribution. Finally, the mean of the resulting distribution is used to define the background parameter *b* of a membrane.

As shown in Fig. 3, the background values *b* exhibit a linear relationship to the exposure time. Moreover, the deviation of the distribution *σ*_*b*_ grows linear over time.

### Reaction kinetic

A commonly used detection method in membrane arrays is based on light generated by the enzyme horseradish peroxidase which reacts with H_2_O_2_ to activate luminol. The kinetics of this reaction were extensively studied by Cormier and Prichard in 1968 [4]. Our approach utilizes this knowledge as the foundation to model the light production. The reaction steps of luminol (LH_2_) with H_2_O_2_ catalyzed by the horseradish peroxidase enzyme (E) are listed in Eqs. 4 to 7.

4 $$\begin{array}{*{20}l} {\mathrm{E}} + {{{\mathrm{H}}_{2}}{{\mathrm{O}}_{2}}} &\longrightarrow {{\mathrm{C}}_{\mathrm{I}}}  \end{array} $$

5 $$\begin{array}{*{20}l} {{\mathrm{C}}_{\mathrm{I}}} + {{\text{LH}}_{2}}&\longrightarrow {{\mathrm{C}}_{\text{II}}} + {\text{LH}.} \end{array} $$

6 $$\begin{array}{*{20}l} {{\mathrm{C}}_{\text{II}}} + {{\text{LH}}_{2}}&\longrightarrow {E} + {\text{LH}.} \end{array} $$

7 $$\begin{array}{*{20}l} {2 \text{LH}.} + {{{\mathrm{H}}_{2}}{{\mathrm{O}}_{2}}}&\longrightarrow {h\nu} + {\text{products}}  \end{array} $$

The reaction rate (*v*) can be simplified and written as in Eq. 8. Here, the reaction constants presented by Cormier and Prichard [4] are combined into the parameters *α*,*β*, and *γ* for clarity.

8 $$ v=\frac{\alpha {C}_{{E}}C_{{\text{LH}}_{2}}C_{{\mathrm{H}_{2}}{\mathrm{O}_{2}}}}{\beta C_{{\text{LH}}_{2}}+\gamma C_{{\mathrm{H}_{2}}{\mathrm{O}_{2}}}}   $$

Using the numeric results published by Cormier and Prichard [4] and Chance [9], we can deduct the relation of the simplified parameters to be *α*>*γ*>*β*. Additionally, an excess of H_2_O_2_ ($\phantom {\dot {i}\!}C_{{\mathrm {H}_{2}}{\mathrm {O}_{2}}} > C_{{\text {LH}}_{2}}$) is expected, resulting in a further simplification represented in Eq. 10

9 $$\begin{array}{*{20}l} v&=\frac{\alpha {{C}_{\mathrm{E}}}{{C}_{{\text{LH}}_{2}}}{{C}_{{\mathrm{H}_{2}}{\mathrm{O}_{2}}}}}{\gamma {C}_{{\mathrm{H}_{2}}{\mathrm{O}_{2}}}} \end{array} $$

10 $$\begin{array}{*{20}l} v&={{k}_{\mathrm{r}}}{{C}_{\mathrm{E}}}{{C}_{{\text{LH}}_{2}}} \end{array} $$

The value of the apparent rate constant (*k*_r_=*α*/*γ*) can be approximated to *k*_r_≈1.4×10^6^(Ms)^−1^. As the apparent rate constant *k*_r_, and the concentration of the catalytic enzyme *C*_E_ are independent of the reaction time, the reaction rate development is determined by the concentration of one reagent (LH_2_). Under these circumstances, the reaction can be described as of first order and Eq. 11 outlines the resulting evolution of the luminol concentration. The reaction rate of the enzyme can be expressed in a time-dependent manner (Eq. 12).

11 $$\begin{array}{*{20}l} C_{{{\text{LH}}_{2}}}(t) &= C_{0, {{\text{LH}}_{2}}} \exp{(-k_{\mathrm{r}}C_{\mathrm{E}}t)}  \end{array} $$

12 $$\begin{array}{*{20}l} v(t)&=k_{\mathrm{r}}C_{\mathrm{E}}C_{0, {{\text{LH}}_{2}}} \exp{(-k_{\mathrm{r}}C_{\mathrm{E}}t)} \end{array} $$

A light flux is produced based on the reaction turnover (Eq. 7) and its intensity is related to the reaction speed *v*(*t*)∝*I*(*t*). By introducing the proportionality factor *k*_i_, we can describe the light intensity *I*(*t*) in Eq. 13. The variable *t* is chosen to represent the exposure time as recorded by the image acquisition device, to enable better comparison with the experimental data. The time between starting the reaction (i.e., pipetting the reagent onto the membrane) and the start of the image recording is included by adding the preparation time *t*_0_ as a constant.

13 $$\begin{array}{*{20}l} I(t)&=k_{\mathrm{i}}k_{\mathrm{r}}C_{\mathrm{E}}C_{0, {{\text{LH}}_{2}}} \exp{\left(-k_{\mathrm{r}}C_{\mathrm{E}}(t+t_{0})\right)} \end{array} $$

In the experimental setup, the light flux is measured cumulative over time. Therefore, Eq. 13 is integrated over the exposure time to analyze the data.

14 $$\begin{array}{*{20}l} \int^{t}_{0}\!\!I(t)\,\mathrm{d}t&=k_{\mathrm{i}}C_{0, {{\text{LH}}_{2}}} \left[-\exp{(-k_{\mathrm{r}}C_{\mathrm{E}}(t_{0}+t))}\right.\\ &\qquad\qquad\quad\left.+\exp{\left(-k_{\mathrm{r}}C_{\mathrm{E}}t_{0}\right)}\right] \end{array} $$

15 $$\begin{array}{*{20}l} L(t)&=\kappa \,\left[-\exp{\left(-k_{\mathrm{r}}C_{\mathrm{E}}(t_{0}+t)\right)}\right.\\ &\qquad\quad\left.+\exp{\left(-k_{\mathrm{r}}C_{\mathrm{E}}t_{0}\right)}\right] \end{array} $$

The solution for this integration in the boundaries is presented in Eq. 14. As the prefactors will be indistinguishable in the analytics of the data, they are combined as $\kappa = k_{\mathrm {i}}C_{0, {{\text {LH}}_{2}}}\phantom {\dot {i}\!}$ (Eq. 15) to simplify the expression. This factor *κ* is specific for each image set. It depends on the initial luminol concentration, on the emission efficiency, and the specifics of the recording equipment.

**Fig. 4:**
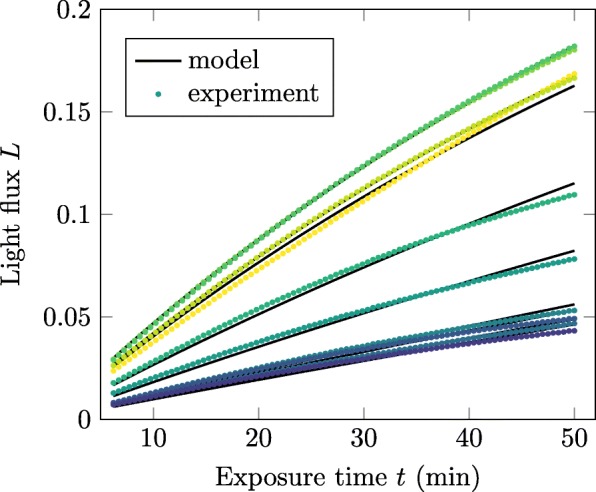
Determine *κ*. Result of a simultaneous fit of the ten brightest spots on a membrane to extract the prefactor *κ*

In the implementation of *proMAD*, the light flux *L*(*t*) is measured by the average signal in the square around each spot reduced by the background value *b* of that frame. The simplified prefactor *κ* is defined by minimizing the errors of the brightest spots simultaneous while fitting to Eq. 15 (see Fig. 4). Finally, the concentration of the horseradish peroxidase enzyme *C*_E_ bounded to the proteins is determined with the estimated *κ* for each spot individually. The target protein content bound on the membrane is directly reflected by the enzyme concentration.

## Results and discussion

The core algorithm of *proMAD* can be accessed through the open-source library or the web application. The Python library can be implemented in user-specific analysis setups and presents a wide range of opportunities for customization. The web tool is suited for users who want to run the stable version *proMAD* without the need to install any software.

At present, a range of commonly used image formats can be imported. Raw images (.scn) from ChemiDoc™ MP systems (BioRad, Gladesville, Australia) can be used directly. The core algorithm, the reaction-based model, is available when the exposure times can be accessed in the loaded images. Multiple additional evaluation modes can be selected in cases where the exposure time information is missing. The raw mode returns a list of averaged gray values for the spots on all original images in the stack. Deduction of the histogram-based background value from this list is possible. Moreover, a morphological background detection can be used to separate the foreground and the background of the raw images. The local background approach calculates the mean of the ratios between the average of a spot on the raw images, and the extracted backgrounds. Besides, the linear correlation between the histogram background value and the average foreground value or the raw image over time can be used to evaluate the signals. The results for a selected evaluation method can be summarized in a report. At present, four report modules are available: json, csv, excel, and LATE X. The excel and LATE X report files contain the average values of each analyte, as well as a graphical representation of the samples with the highest signals. An image of the membrane serves as an alignment check. Additionally, information about the software version and user-provided naming of the data set and membranes is included in the report. Currently, *proMAD* supports four different membrane types. However, other membrane array layouts can be easily implemented, given the presence of at least three reference spots for the alignment algorithm.

Fundamental aspects of the toolkit, such as the algorithm to measure the intensity as well as the automatic alignment system and the background analysis can be adapted for other types of densitometric measurements. The main algorithm presented, depends on the reduction of the light-emitting material over time. Therefore, it can be applied to techniques that are also using an enzyme-based detection method with known reaction kinetics.

### Library

The *proMAD* library is accessible through the Python Package Index (PyPI). Alternatively, it can be installed directly using the code available on Github [10]. The user can set up a highly tailored workflow and integration with other analysis protocols.

### Web application

The *proMAD* web application [11] provides an easy-to-use interface to process membrane images in the cloud. This approach is suitable to analyze small sample sets without the need to set up the development environment. The web interface guides the users through the process step-by-step. Multiple input files can be bundled in a zip or tar container to accelerate the upload. Several membranes recorded in one image stack can be separated and processed. The process can be completed in multiple sessions using the displayed request key. At the end of the image analysis process, various formats for downloading the results are presented.

## Conclusion

Here we present *proMAD*, a novel systemic analytical approach for the quantification of membrane protein arrays. The distinctive, self-referencing concept is based on the evaluation of the dynamic signal development using the knowledge about the kinetics of the underlying chemical reaction. Modern image acquisition devices allow for collecting data about signal development over time for each given membrane. By utilizing this information, deviations between different membranes can be accounted for, enabling better comparison of such within a given experimental setup. While the web application provides an easy and platform-independent access to the *proMAD* core algorithm to a wide range of researchers, the underlying open-source library, on the other hand, allows for highly customized data analysis workflows. The toolkit is relevant to a variety of end-users while keeping the underlying algorithm openly accessible. *proMAD’s* intrinsic flexibility has the potential to apply the presented algorithm to a wide range of additional use-cases.

## Availability and requirements

Project name: proMAD Project home page: https://promad.devSource code: https://github.com/theia-dev/proMADOperating system(s): platform-independent; web application Programming language: Python ≥3.6 Other requirements: refer to requirements.txtLicense: MITAny restrictions to use by non-academics: None

## Supplementary information

**Additional file 1** Supplementary information. The supplementary file contains the detailed experimental procedures for obtaining the processed images.

## Data Availability

Not applicable.

## Abbreviations

*b*: Background level
*b*_G_: Background level guess
*C*: Concentration
*C*_0_: Initial concentration
*C*_X,Y_: Contour center
E: Horseradish peroxidase enzym
*G*: Anchor point guess
*h*: Planck constant
*I*: Light intensity
*k*_i_: Proportionally constant
*k*_r_: Apparent rate constant
*L*: Light flux
LH_2_: Luminol
*t*: Time
*t*_0_: Recording start time
*v*: Reaction speed
I,II: Intermediate reaction products
*α*,*β*,*γ*: Reaction parameter
*σ*_*b*_: Background level standard deviation
*κ*: Combined prefactor
*ν*: Photon frequency
