# Supplementary material for: proMAD: semiquantitative densitometric measurement of protein microarrays
Source: BMC Bioinformatics. 2020 Feb 24;21:72. doi: 10.1186/s12859-020-3402-4 (PMC7041094; doi:10.1186/s12859-020-3402-4)
Supplement: Supplementary file 1 — Additional file 1 Supplementary information. The supplementary file contains the detailed experimental procedures for obtaining the processed images. [file 12859_2020_3402_MOESM1_ESM.pdf]

SOFTWARE

proMAD: semiquantitative densitometric measurement of protein microarrays

Additional file 1 — Supplementary Information

Anna Jaeschke[1,2†], Hagen Eckert[3,4*†] and Laura J Bray[1,2]

*Correspondence:
hagen.eckert@tu-dresden.de
[3]Institute for Materials Science and Max Bergmann Center of Biomaterials, Technische Universität Dresden, Dresden, Germany
Full list of author information is available at the end of the article
†Equal contributor

Experimental Procedures

Cell culture

Cells were cultured in starPEG-heparin hydrogels as described before [1].

Protein Isolation

Hydrogels were briefly washed in PBS and incubated with $1\,\mathrm{mg\,ml^{-1}}$ Collagenase NB 4G (Serva Electrophoresis, Heidelberg, Germany) at 37 °C for 20 min. Additional disruption was achieved using a P1000 three times during the incubation. The collagenase digestion was stopped by the addition of 2 mM EDTA/PBS (Sigma-Aldrich, Castle Hill, Australia) in a 1:1 ratio. The cells were pelleted by centrifugation at 300 rcf for 10 min. Prior to lysis, the cell pellet was washed two times with PBS to remove traces of collagenase. Lysis of the cells was achieved by resuspending the pellet in 70 µL RIPA buffer (Sigma-Aldrich, Castle Hill, Australia) containing a Protease/Phosphatase Inhibitor Mix (ThermoFisher, Scoresby, Australia). The lysate was kept on ice for 15 min, during which the samples were mixed with a vortex three times for 30 sec, and then stored at −80 °C.

Whole Protein Content Quantification

Pierce™ BCA Protein Assay Kit (ThermoFisher, Scoresby, Australia) was used according to the manufacturer's protocol for limited sample size to quantify whole protein content in the samples. The protein content was determined using a calibration curve of known protein concentrations ranging between $25\,\mathrm{\mu g\,\mu L^{-1}}$ to $2000\,\mathrm{\mu g\,\mu L^{-1}}$. Extrapolation of unknown samples was achieved using the drc package in R [2, 3].

Proteome Profiler Kit

Human XL Cytokine Array Kit, ARY022B, (R&D Systems, Minneapolis, USA) was used according to manufacturer's protocol. The total protein content used per membrane was kept equal between samples. Briefly, the cell lysate was incubated with the membrane overnight. A Streptavidin-Horseradish peroxidase conjugate and chemiluminescent detection reagent were applied for immunodetection of captured proteins.

Image Acquisition

Images of membranes were acquired using a ChemiDoc™ MP system (BioRad, Gladesville, Australia). For each set of membranes, 100 images were taken, the first after 10 sec and the last after 3000 sec with binning set to 2 x 2. Subsequently, the same set of membranes was imaged with binning set to 4x4 and 100 images taken between 10 sec and 1200 sec. One set of membranes contained 4 or 5 membranes which were imaged in one image stack.

Author details

[1]Institute of Health and Biomedical Innovation, Queensland University of Technology, 4059 Kelvin Grove, Queensland, Australia. [2]School of Mechanical, Medical and Process Engineering, Science and Engineering Faculty, Queensland University of Technology, 4059 Kelvin Grove, Queensland, Australia. [3]Institute for Materials Science and Max Bergmann Center of Biomaterials, Technische Universität Dresden, Dresden, Germany. [4]Dresden Center for Computational Materials Science (DCMS), Technische Universität Dresden, Dresden, Germany.

References

1. Tsurkan, M.V., Chwalek, K., Prokoph, S., Zieris, A., Levental, K.R., Freudenberg, U., Werner, C.: Defined Polymer-Peptide Conjugates to Form Cell-Instructive starPEG-Heparin Matrices In Situ. Advanced Materials **25**(18), 2606–2610 (2013). doi:10.1002/adma.201300691
2. Ritz, C., Baty, F., Streibig, J.C., Gerhard, D.: Dose-Response Analysis Using R. PLOS ONE **10**(12), 0146021 (2015). doi:10.1371/journal.pone.0146021
3. R Core Team: R: A Language and Environment for Statistical Computing. R Foundation for Statistical Computing, Vienna, Austria (2019)
